# Where to from here? Identifying and prioritising future directions for addressing drug-resistant infection in Australia

**DOI:** 10.1186/s13756-021-00953-4

**Published:** 2021-05-29

**Authors:** Gregory Merlo, Minyon Avent, Trent Yarwood, Bonnie Smith, Mieke van Driel, Lisa Hall

**Affiliations:** 1 Primary Care Clinical Unit, Faculty of Medicine, Royal Brisbane & Women’s Hospital, University of Queensland, Level 8 Health Sciences Building, Building 16/910, Brisbane, QLD 4029 Australia; 2 School of Public Health, Faculty of Medicine, University of Queensland, Brisbane, Australia; 3 UQ Centre for Clinical Research, Faculty of Medicine, University of Queensland, Brisbane, Australia; 4 Queensland Statewide Antimicrobial Stewardship Program, Brisbane, Australia; 5 Cairns Clinical School, College of Medicine and Dentistry, James Cook University, Douglas, Australia; 6 Rural Clinical School, Faculty of Medicine, University of Queensland, Brisbane, Australia

**Keywords:** World café, Antimicrobial stewardship, Antibiotic resistance, Implementation, Forum

## Abstract

**Background:**

The Australian National Antimicrobial Resistance Strategy calls for a collaborative effort to change practices that have contributed to the development of drug-resistance and for implementation of new initiatives to reduce antibiotic use.

**Methods:**

A facilitated workshop was undertaken at the 2019 National Australian Antimicrobial Resistance Forum to explore the complexity of antimicrobial stewardship (AMS) implementation in Australia and prioritise future action. Participants engaged in rotating rounds of discussion using a world café format addressing six topics relating to AMS implementation. Once all tables had discussed all themes the discussion concluded and notes were summarised. The documents were independently openly coded by two researchers to identify elements relating to the implementation of antimicrobial stewardship.

**Results:**

There were 39 participants in the facilitated discussions, including pharmacists, infectious disease physicians, infection prevention nurses, and others. Participants discussed strategies they had found successful, including having a regular presence in clinical areas, adapting messaging and implementation strategies for different disciplines, maintaining positivity, and being patient-focused. Many of the recommendations for the next step involved being patient focussed and outcomesdriven. This involves linking data to practice, using patient stories, using data to celebrate wins and creating incentives.

**Discussion:**

Recommendations from the workshop should be included in priority setting for the implementation of AMS initiatives across Australia.

**Supplementary Information:**

The online version contains supplementary material available at 10.1186/s13756-021-00953-4.

The Australian National Antimicrobial Resistance Strategy calls for a collaborative effort to change practices that have contributed to the development of drug-resistance and for implementation of new initiatives to reduce antibiotic use [1]. In Australia, achievements have included an expanded role of hospital pharmacists in supporting appropriate antibiotic prescribing—now mandated in national accreditation standards [2, 3], improvements in surveillance, including the introduction of the Antimicrobial Use and Resistance in Australia (AURA) Surveillance System [4, 5]; the use of electronic referral applications for audit and feedback rounds [6], and the introduction of processes for incorporating antimicrobial stewardship into discharge [7]. Nevertheless, progress more broadly has been slow and novel solutions are now required for improving clinical practice and community awareness in a sustainable fashion.

A facilitated workshop was undertaken at the 2019 National Australian Antimicrobial Resistance Forum to prioritise future directions for antimicrobial stewardship implementation in Australia. The forum brought together health professionals, veterinarians, policy makers, and others to promote effective action against antimicrobial resistance. Participants (n = 39)—including health professionals, veterinarians, policy makers, and others—engaged in rotating rounds of discussion using a world café format addressing six topics relating to AMS implementation (see Fig. 1) [8]. Once all tables had discussed all themes the discussion concluded, and notes were summarised. The documents were independently openly coded by two researchers to identify elements relating to the implementation of antimicrobial stewardship. An iterative approach was used to identify and reach consensus on emergent themes from the workshop. A summary of results was sent out to all workshop participants for feedback (see Additional file 1).

**Fig. 1:**
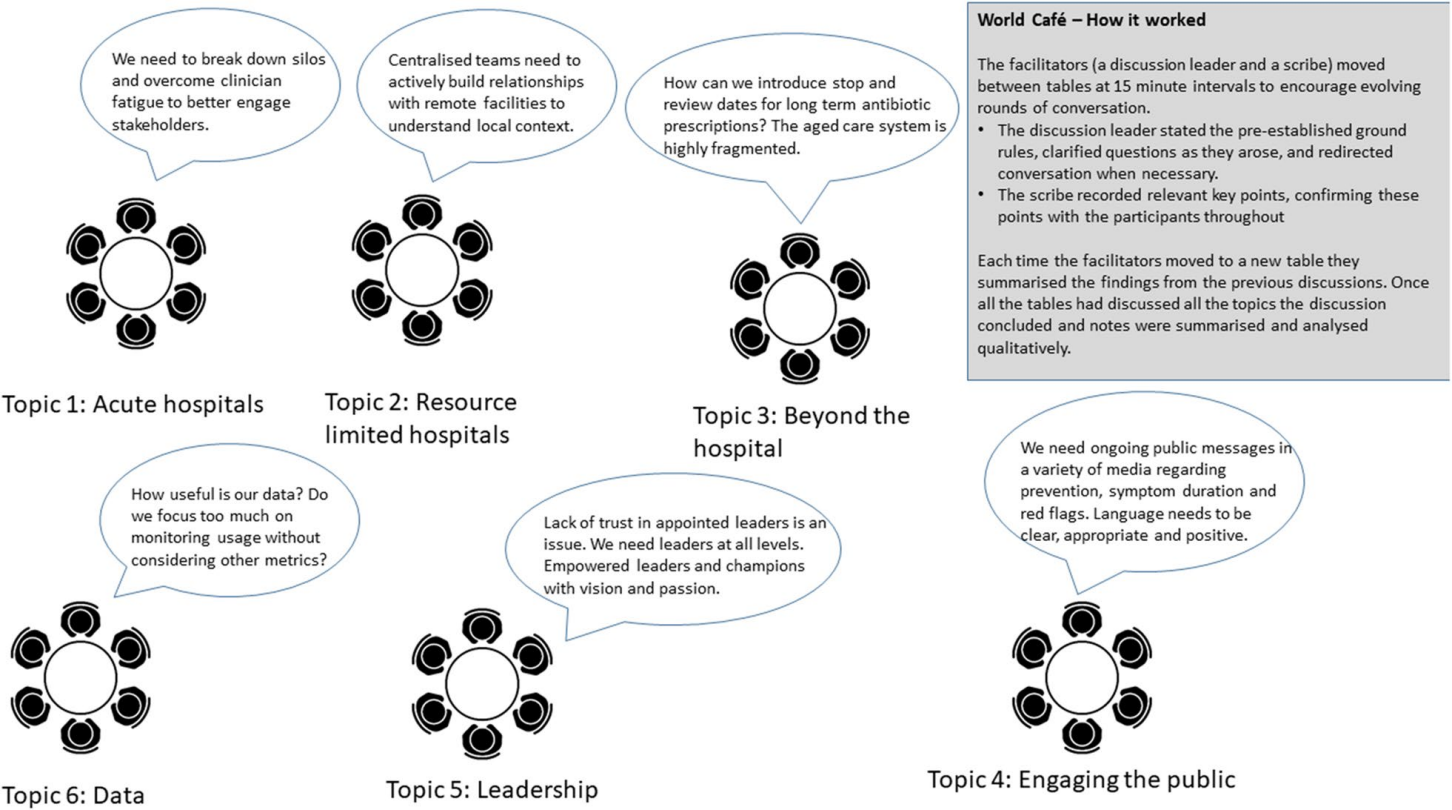
World café method

## Topic 1: building rapport within hospital setting

Successful strategies observed by participants for building rapport within a hospital setting included having a regular presence in clinical areas, adapting messaging and implementation strategies for different disciplines and different levels of seniority, maintaining positivity, and being patient focused. Maintaining a priority status for AMS compared with other needs in a hospital setting is difficult. It can result in what one participant called “AMS fatigue”, where individuals becoming weary of hearing about the problem and so interest in AMS cannot be maintained. Recommendations for the next step involved being patient focussed and outcomes driven, linking data to practice, using patient stories, and using data to celebrate wins and creating incentives.

## Topic 2: implementing in a resource limited hospital setting

Reported challenges in implementing AMS within a resource limited hospital setting, such as rural and remote hospitals, include lack of access to relevant expertise, burnout of healthcare workers, a high rate of staff turnover, and the most appropriate antibiotic as recommended by Therapeutic Guidelines not always being available [9] due to lack of supply. Participants suggested a centralised service to be formalised and resourced appropriately that would work to build relationships with the facilities and understand the local context. One of the roles of such a centralised service would be to provide education—whether remotely or in-person—to build local expertise.

## Topic 3: implementing beyond the hospital setting

The groups discussed implementation of AMS beyond the hospital setting, including general practice, community pharmacy, and aged care. Participants identified that despite the differences in each of these settings they have shared challenges of fragmentation and misaligned incentives, and lack of access to services that may be available in a hospital setting. There are time pressures compounded by patient expectations of receiving antibiotics—it can seem faster and easier to “just prescribe”. Within community pharmacies, barriers to AMS include lack of financial motivation to minimise antibiotic use and changing business models leading to some pharmacies providing less patient support. Specific concerns in the aged care sector included high turnover of staff, widespread polypharmacy, and continuity of care, and the need for greater access to specialist AMS support. Participants discussed training, peer support and surveillance with feedback as strategies for implementing AMS in the community setting. The participants argued for the potential value of general practitioners and pharmacists having access to the same data.

## Topic 4: engaging and empowering the public

Participants identified that there are challenges with health literacy regarding prevention of infections, appropriate treatments, and duration of symptoms. They argued that AMS needs to be reframed to engage and empower the public. They recommended using positive language with individualised messages to patient groups establishing expectations for patients and providing patients with strategies to manage colds and flus. The messaging to patients should be consistent across professional groups to minimise patient confusion and be available across multiple media.

## Topic 5: leadership

Participants discussed how to develop and encourage AMS leadership at all levels and the role of AMS leadership organisations both nationally and state-wide. One of the challenges identified was the lack of role clarity between the different AMS leadership organisations, making it difficult to know who is responsible for what task and how to provide oversight for these organisations.

## Topic 6: linking data with implementation strategies

The absence of funding, time, and expertise were identified as challenges with linking implementation strategies to data. It can be difficult to determine if differences in antibiotic prescribing are due to variation in clinical case mix rather than differences in the prescribing behaviour of clinicians. Good quality and relevant data is not sufficient; the data also needs to be disseminated—whether this be to executives, health professionals, or the public. Participants recommended that data be presented in a way that tells a story, considers cost-effectiveness, and highlights benefits to the patient, such as linking to patient outcomes.

## Supplementary Information

**Additional file 1**. Summary of reflections on current progress and priorities identified.

## Data Availability

The data that support the findings of this study are available from the corresponding author (GM) upon reasonable request.
