# Supplementary material for: Where to from here? Identifying and prioritising future directions for addressing drug-resistant infection in Australia
Source: Antimicrob Resist Infect Control. 2021 May 29;10:85. doi: 10.1186/s13756-021-00953-4 (PMC8164738; doi:10.1186/s13756-021-00953-4)
Supplement: Supplementary file 1 — Additional file 1. Summary of reflections on current progress and priorities identified. [file 13756_2021_953_MOESM1_ESM.docx]

# **Supplement A: Summary of reflections on current progress from antimicrobial stewardship workshop**

#### Topic 1: Engagement strategies and building rapport to promote AMS in the hospital setting

| **What has worked?** | **What have been the barriers?** |
| --- | --- |
| - Regular presence - Engagement (top level) - Feedback - AMS champion (mentor) - Adaptation for different disciplines - Positive culture; emotion and empowerment - Interventions: data driven and syndromic based | - Competing priorities - Siloed thinking - Lack of engagement of non-ID staff - Lack of IT support - Rotational AMS teams (lack of consistency) - AMS fatigue |

#### Topic 2: Implementing and sustaining AMS in resource limited hospital settings

| **What has worked?** | **What have been the barriers?** |
| --- | --- |
| - Access to local guidelines - Access to experts who understand local context - Strategies that are linked to core functions such as medication safety - Telehealth - Executive support; local ownership of the AMS program - Support of local people - AMS champions (healthcare workers other than ID/clinical microbiologist) - Education to build local expertise | - Lack of access to expertise - Lack of formal arrangements - Centralised teams may not understand local context – resources, access, indigenous populations - Burn out of healthcare workers; high turn-over rate of staff - Competing priorities - Lack of executive support - Timely access to the right antimicrobial in remote locations - Private hospitals bring in third party pharmacy providers – budget constraints |

#### Topic 3: Implementing and sustaining AMS resources beyond the hospital setting

| **What has worked?** | **What have been the barriers?** |
| --- | --- |
| - Regular peer feedback - Engagement with practice nurses | - Fragmented care. Aged care provided by different off-site GPs - Accessibility to data including microbiology results; - No onsite access to electronic Therapeutic Guidelines in many sites - Limited time for patient consultations - Diagnostic uncertainty - Aged care nurses advocating for patients, believing that antibiotics are needed for asymptomatic bacteriuria and upper RTI, and applying pressure on the GP for a prescription - Few rapid point of care tests; don’t fit in work flow of general practice - Pharmacy guild want to introduce over the counter antibiotics, but pharmacists are not set up or trained for diagnosis. - Fear of patients going elsewhere to get antibiotics - Community pharmacists income stream is dependent on product supply (dispense or OTC). There is not only an absence of reward for preventing or denying supply, but a perverse incentive to supply even if not appropriate |

#### Topic 4: Engaging and empowering the public

| **What has worked?** | **What have been the barriers?** |
| --- | --- |
| - Campaigns: raised awareness National Prescribing Service – - Repetition vs saturation | - No government or community processes for AMS - Language need to be clear and appropriate; Don’t use “AMS” acronym - Lot of misconceptions - Message needs to be balanced, including both benefits and harm - No government or community processes for AMS - Poor health literacy regarding prevention of infections, appropriate treatments and duration of symptoms - Lack of funding - Changing model of pharmacy; less patient support in a pharmacy chain - Anti-vaccination information readily available - Availability of antibiotics over the internet |

#### Topic 5: Leadership

| **What has worked?** | **What have been the barriers?** |
| --- | --- |
| - Good model: State-wide coordinated programs e.g. NSW Clinical Excellence Commission - Leaders at all levels: UN’s Interagency Coordination Group on antimicrobial resistance - Australian Commission on Safety and Quality in Healthcare providing oversight | - Many overlapping organisations (most people don’t know who is responsible for what) - Lack of clarity about the roles and responsibilities at all levels - Multiple different levels of funding and accountability – private vs public - Lack of accreditation standards for AMS in primary care - Inconsistent/mixed messages (takes time to educate our leaders) - Who ‘polices’ the police (oversight of ID physicians & AMS programs) - Lack of ownership; engagement and sustainability of leaders - Lack of trust in our leaders - Politicians follow public opinion (AMS not perceived as a priority) |

#### Topic 6: Linking data with implementation strategies

| **What has worked?** | **What have been the barriers?** |
| --- | --- |
| - National Antimicrobial Utilisation Surveillance Program - Antibiograms (need to filter data) - Peer comparison of data (limited due to variation between facilities) | - How meaningful is our data (do we focus too much on usage without considering other resources?) - What are useful metrics? DDD (increasing focus on PK/PD e.g. obesity) vs DOT - Time (volume of data required) & expertise required to collect and analysis data - Dissemination of information (executive; healthcare workers & public) - Lack of information from the community - Interventions have not used behaviour change principles - Lack of funding |

AMS, antimicrobial stewardship; DDD, defined daily dose; DOT, days of therapy; GP, general practitioner; ID, infectious disease; NSW, New South Wales; OTC, over the counter; PD, pharmacodynamics; PK, pharmacokinetics; RTI, respiratory tract infection; UN, United Nations

# **Supplement B: Priorities identified at national antimicrobial stewardship workshop**

| **Topic** | **What should we do next** |
| --- | --- |
| Engagement strategies and building rapport to promote AMS in acute hospitals | - Celebrate wins - Greater collaboration with non-AMS staff (e.g. nurses) - Incentives - Rebrand AMR/AMS - Overcome clinician fatigue and silos - Non-antibiotic interventions (prevention) |
| Implementing and sustaining AMS in resource limited hospital settings | - Links to centralised services need to be formalised and resourced appropriately - Centralised team needs to actively work to build relationships with the facilities, understand local context - Establishing connections across private & public sectors - Empower skilled nurses - Include Aboriginal healthcare workers in AMS initiatives - Make AMS everyone’s problem |
| Implementing and sustaining AMS resources beyond the hospital setting | - Collect and share data on patient outcomes - Engagement through GP champions - Greater access to specialist support - Credentialing for prescribing antibiotics - Education settings: including school programs - Healthcare professionals (doctors, pharmacists etc) to have access to the same data - Greater access to specialist support - Credentialing for prescribing antibiotics - Accreditation linked to framework such as hospital setting - Education: including school programs, patient information leaflets etc - Aged care: point of care tests, process for de-prescribing - Process for de-prescribing in aged care - Introducing stop dates for long term antibiotics - Incentives through Medicare reimbursement - Accreditation linked to framework like in hospital settings |
| Engaging and empowering the public | - Improve communication between healthcare professionals so there is a consistent message - Engagement should be interesting and relevant - Language – use positive language (avoid scaremonger) - Individualised messages to patient groups - Focus on consumer behaviour (manage fear) - Need ongoing public messages in a variety of media regarding prevention, symptom duration and red flags - Greater focus on patient stories |
| Leadership | - Political engagement - Engagement with organisations at a national level - Leaders with vision; passion & empowerment - Leaders and champions at all levels (amongst your peers; non-traditional) |
| Linking data with implementation strategies | - Use measures of appropriateness rather than usage (more relevant for public) - Better patient outcome data needed - Measure top 10 antibiotics prescribed - Use health economics methods to evaluate implementation strategies - How should data be analysed   - Needs to tell a story   - Link resistance data and cleaning   - Link usage (including antibiotic shortages) and resistance at a facility level   - Link antibiotics and side effects   - Assess duration versus harm |

AMR, antimicrobial resistance; AMS, antimicrobial stewardship
